# Environmental Enrichment Devices Are Safe and Effective at Reducing Undesirable Behaviors in California Sea Lions and Northern Elephant Seals during Rehabilitation

**DOI:** 10.3390/ani13071222

**Published:** 2023-03-31

**Authors:** Kirsten Donald, Amanda Benedetti, Vinícius Donisete Lima Rodrigues Goulart, Alissa Deming, Hendrik Nollens, Grey Stafford, Sabrina Brando

**Affiliations:** 1 Pacific Marine Mammal Center, Conservation Medicine and Science, Laguna Beach, CA 92651, USA; 2 Transportation Research and Environmental Modelling Lab—TREM, Institute of Geosciences, Universidade Federal de Minas Gerais, Belo Horizonte 31741-460, Brazil; 3 San Diego Zoo Wildlife Alliance, San Diego, CA 92112, USA; 4 KONG™ Zoo, Golden, CO 80403, USA; 5 AnimalConcepts, 03725 Teulada, Spain

**Keywords:** rehabilitation, pinnipeds, environmental enrichment, animal welfare, stereotypic behavior, habituation

## Abstract

**Simple Summary:**

The use of enrichment in zoos and aquariums has a positive effect on the wellbeing of animals, and supports their reintroduction success; however, research concerning their use in rehabilitation centers is still limited. This study investigated three different enrichment devices and their ability to decrease undesired repetitive and/or anticipatory behaviors in California sea lions and northern elephant seals undergoing rehabilitation in Southern California. All three devices were found to be durable and safe throughout the study, and demonstrated a significant reduction in animals displaying undesirable stereotypical behaviors compared to observations when no enrichment devices were used. This study highlights the beneficial aspect of environmental enrichment devices for pinnipeds hospitalized in rehabilitation centers.

**Abstract:**

Environmental enrichment devices (EEDs) have been proven to promote positive wellbeing in zoos and aquariums, and support animals’ reintroduction success; however, their use in rehabilitation centers is still limited. This pilot study investigated the safety and efficacy of three EEDs, Artificial Kelp, Horse KONG^™^ and Wubba Kong^™^, and their ability to decrease and/or eliminate undesired stereotypic behaviors or looking at staff/staff areas in seven wild California sea lions (CSLs) and eight northern elephant seals (NESs) undergoing rehabilitation in Southern California. Observers conducted instantaneous sampling once a minute during a 30-min baseline, followed by a 30-min EED implementation on one focal animal at a time. The data were analyzed with generalized linear mixed models (GLMM). All three EEDs were found to be durable and safe throughout the study. Our results show a significant reduction in stereotypical behaviors compared to no EED treatments in CSLs, with the most significant effect being with the Horse KONG^™^. The Wubba KONG™ and Artificial Kelp provisions also reduced the undesired behavior in both species of being focused on human caretakers. Individual preferences for specific EEDs were found within species and between species, with the NESs using EEDs more than the CSLs. This study highlights the beneficial aspect of EEDs for pinnipeds in rehabilitation centers for improving their quality of life.

## 1. Introduction

Modifications that act to enhance captive environments are referred to as environmental enrichment [1]. The Association of Zoos and Aquariums defines enrichment as “a process for improving or enhancing zoo animal environments and care within the context of their inhabitants’ behavioral biology and natural history”. Environmental enrichment devices (EEDs) have been shown to result in positive outcomes on animal behavior [2], with psychological and physiological benefits [3]. There is a long history of the use of EEDs in zoos [4,5], aquariums [6] and animal shelters [7]; however, the use of EEDs in wildlife rehabilitation settings is limited.

Pinnipeds are globally the most common marine mammal in rehabilitation facilities. On the southern west coast of the United States, the most common species include California sea lions (*Zalophus californianus*), northern elephant seals (*Mirounga angustirostris*) and Pacific harbor seals (*Phoca vitulina*). The primary goal of marine mammal rehabilitation is to treat sick and injured wild animals and release them back to their natural habitats [8,9].

Pinnipeds in Southern California rehabilitation centers tend to be young (pups, weanlings and yearlings) and, due to maternal separation or challenges during weaning, can encounter health issues such as malnutrition [9], pneumonia, sepsis or injuries [10,11]. Life in the wild exposes young pinnipeds to a variety of stimuli required to develop necessary survival skills, while rehabilitation settings tend to have fewer opportunities to learn these types of species-specific behaviors [12,13]. The introduction of EEDs can help avoid or diminish the effects of stress and contribute to maintaining a species’ natural behavioral repertoire [3,14,15]. Different biological considerations, such as disease, temperament and behavior, can influence reintroduction success and the development of important behavioral traits for an animal’s survival post-release, including moving in complex environments, foraging, interacting in social groups and avoiding conflicts with humans [16]. In the wild, pinnipeds have currents to navigate, underwater vegetation to rest in, as well as beaches and rocks on which to haul out. Therefore, a lack of physical, social and cognitive stimuli during rehabilitation may lead to undesired outcomes, such as atypical behavior (chimpanzees: [17]), stereotypic and or pattern-like behaviors (seals: [6,18]), as well as a reduction in overall natural wild-type behaviors [19,20]. EEDs are one of the tools used to support animal wellbeing and the successful reintroduction of wild animals that have undergone rehabilitation. Pinnipeds are highly intelligent [21], and stranding at this developmentally critical life stage supports the need for adequate enrichment. During rehabilitation, these young, wild pinnipeds are prone to being stressed, at risk of habituation and/or development of stereotypical behaviors such as habitual suckling and pattern swimming [6]. Patients recovering from injuries and illnesses may benefit from the physical therapy and mental stimulation associated with EEDs, if offered at the appropriate time in the recovery process.

Enrichment has been shown to increase reintroduction success in a variety of species [22,23]; however, EEDs are not routinely implemented in a large proportion of marine mammal rehabilitation centers, and their beneficial effects are not yet well understood. One study assessing the impact of providing EEDs to hospitalized young harbor seal pups (*Phoca vitulina richardii*) found a reduction in pattern swimming and increased foraging and exploration behaviors. The pups also took longer to successfully feed on their own, with increased expressions of stereotypical behaviors [18]. Hunter et al. (2002) investigated the behavioral effects of enrichment in the outdoor exhibit of seven harbor seals and two gray seals housed at the National Aquarium in Baltimore, USA. Pattern swimming out of sight (animals not being visible during the observation) decreased, while random swimming and exploration behavior increased when enrichment was present. Ultimately, there is evidence to suggest that EEDs are successful in improving behavioral outcomes in both zoo and rehabilitation settings for pinnipeds; however, further study is necessary to enhance our understanding of species-specific differences in the efficacy of EEDs.

There are currently no published studies assessing the benefits of using EEDs with wild California sea lions (CSLs) or northern elephant seals (NESs) while in rehabilitation. Studies of species-specific differences in enrichment efficacy are beneficial for a multitude of reasons; they allow us to tailor rehabilitation programs to the individual species in question with respect to relevant species and individual differences, rather than generalizing results from other taxa under the assumption that the outcomes will be the same. Generalizations can be problematic, resulting in “rules of thumb” which are applied across taxa without understanding the potential positive and negative effects that different types of EEDs may have for individual species [24].

The primary objectives of this study were to determine the safety and interest of wild CSLs and NESs in three EEDs, as well as their efficacy in encouraging natural behaviors. Assessments were also made on the potential for EEDs to decrease stereotypical behaviors (e.g., suckling on other pups or self; pattern swimming) and/or to decrease the tendency toward habituation to humans.

## 2. Materials and Methods

The Pacific Marine Mammal Center (PMMC) is a marine mammal rescue and rehabilitation hospital in Orange County, California, and has cared for thousands of marine mammals since it was founded over 50 years ago. All rescue, rehabilitation and activities conducted in this study were carried out under a Stranding Agreement between the PMMC and the National Oceanic and Atmospheric Administration (NOAA). The aim of this study was to assess the safety and efficacy of three EEDs: (1) Artificial Kelp, (2) Horse KONG^™^ and (3) Wubba KONG^™^, for use with CSL pups/yearlings and NES weanlings undergoing rehabilitation. The three primary objectives were the following: (1) to gauge the interest of wild rehabilitating pinnipeds in the environmental enrichment devices; (2) to assess whether the enrichment devices were safe and durable for use in pinnipeds; and (3) to gain preliminary insight into whether these types of enrichment activities can assist in (a) reducing stereotypic behavior on land or in water, and (b) reducing the amount of time patients spend looking at humans or toward staff areas. Looking for humans is considered a leading indicator of habituation during the animals’ time in rehabilitation.

### 2.1. Animal Management

In this study all sea lions were pups or yearlings, and all elephant seals were weanlings/pups (Table 1). The CSLs in this study (CSL 1–8) were admitted to the PMMC between April 11 to December 6, 2020. All patients presented as underweight (malnutrition) and were dehydrated, and some had additional morbidities including pneumonia, ocular trauma, wounds, and abscesses as described in Table 1. NES patients (NESs 1–6) included in this study were admitted to the PMMC between 8 March to 26 May 2021. The estimated ages of patients were assigned based on straight length, dentition and time of year, with the knowledge that wild NESs in the region have a synchronized birthing season between January and March (NOAA). 

All patients received nutrition and vitamin supplementation, subcutaneous fluid therapy, medications (e.g., antibiotics, pain medication) and medical procedures (e.g., gastroscopy) as needed, directed by the attending veterinarian (AD). Patients were only enrolled in the study when their health was deemed stable, had pool access, and passed their wellbeing examination completed by the AD. All patients studied were successfully rehabilitated and released back to the wild once they were deemed healthy.

### 2.2. Enrichment Devices

The selected EEDs were reviewed by the PMMC’s veterinarians to ensure they did not pose an entanglement or ingestion hazard. All interactions with EEDs were supervised by animal care staff, and the integrity of each device was inspected before and after each session. The EEDs were cleaned following each session by soaking in a 1:10 solution of bleach to freshwater for a minimum of 1 min, and were left to sun dry before subsequent use. Animals with suspected pox lesions were given a separate set of EEDs, as poxviruses can be indirectly transmitted via fomites.

The three EEDs chosen for use in this study were Artificial Kelp, Horse KONG^™^ and Wubba KONG^™^ (Table 2). The Horse KONG^™^ and Wubba KONG^™^ had not previously been documented with pinnipeds in rehabilitation centers. Artificial Kelp has been used with pinnipeds in zoos, aquariums and rehabilitation centers [6,18,25]. 

**Figure 1:**
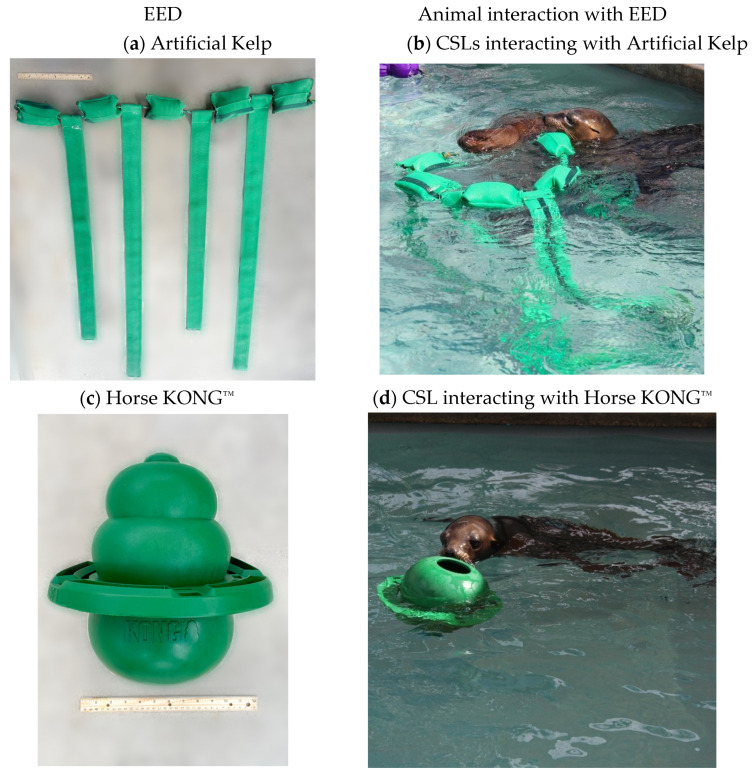

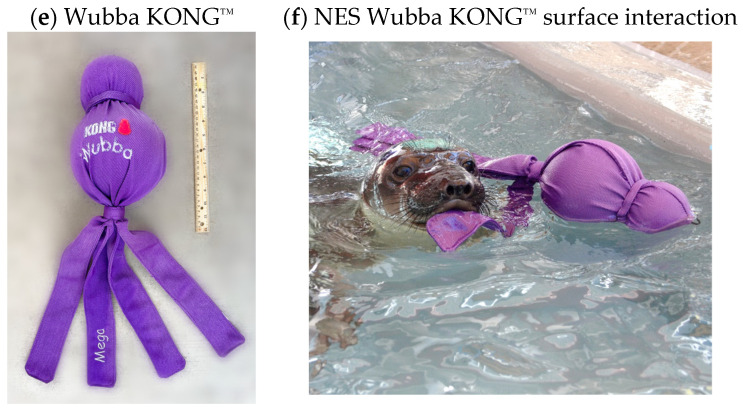
Photos of all EEDs used in this study, and examples of animals interacting with EEDs: Artificial Kelp (**a**,**b**), Horse KONG^™^ (**c**,**d**) and Wubba KONG^™^ (**e**,**f**).

The Artificial Kelp was fabricated from green firehose material provided by KONG^™^ Zoo combined with marine rope and floats. The four strips, two shorter (120 cm) and two longer (155 cm), hang down from the float line. The strips measured 8.5 cm in width. The total width of the kelp was 137 cm, and weighed 1.9 kg (dry). The attachments were created with marine rope with rope floats for buoyancy, and were covered with firehose material (Figure 1). The Horse KONG^™^ measured 30.5 cm in height and 2.0 kg in weight (dry); it was made of a natural rubber blend with an outer ring. The Wubba KONG^™^ consists of one smaller and one larger ball covered with reinforced ballistic nylon fabric and stitching, and four floppy tails, measuring 62 cm in length and 0.7 kg in weight (dry).

### 2.3. Behavioral Sampling

Behavior categories were developed from preliminary live observations: swimming (S); interacting with the enrichment device (IE); social interaction (SI); stereotypic (ST); sleeping (SL); inactive (I); looking at people/care staff area (L); and other (O) (Table 2). Three experienced observers with expertise in animal behavior were involved in the development of the ethogram and collection of behavioral data. Preliminary data collection was conducted on a non-study animal until the consensus between observations for all three observers was 100%. These initial three experienced observers trained eight additional observers on data collection. During the training, one experienced observer was paired with an inexperienced observer, and both recorded observed behaviors for the same animal. If discrepancies were identified, a consensus was determined; this process was repeated until the trainee and experienced observer recorded 100% consistent behaviors for 2 or more observation periods. Once this was achieved, the trainee could independently observe during the data collection period. The observers conducted instantaneous sampling once a minute during a 30-minute baseline followed by a 30-minute EED implementation with 1 focal animal at a time, also using instantaneous sampling once a minute [26].

Three different EEDs were individually presented to each animal in a random sequence, on different days. Each EED type was presented three times, resulting in a total of nine observations per animal (see Table 3). All EED sessions were preceded by a session with no enrichment, which was used to characterize baseline behaviors and control treatment. Individuals participated in one experimental session per day. The observations were performed during the day, and EED sessions never overlapped with feeding times. The patients stayed at the PMMC until they were ready for release, and no releases were delayed for the purposes of the study, resulting in an unequal block design for EED presentation. For three CSL patients in the study, Julius, Pigeon, and Pompey, one session with Artificial Kelp was excluded from the analysis, as the EED was removed early due to unexpected behavioral responses which were deemed unacceptable for the wellbeing of the animals in that moment.

### 2.4. EED Introduction and Removal

After a thirty-minute baseline observation (control session), the selected EED was introduced into the animal’s pool. No introduction of any of the EEDs was carried out prior to the start of the study. An animal care team member would enter the enclosure, placing the EED into the water and exiting. A baffle board was used to protect animal care staff from the animals. Once the observations were completed, an animal care team member would re-enter the enclosure to remove the EED. If the animal was interacting with the EED at the time of entry, the team member would wait to remove the device, in order to not take it from the animal during interaction. None of the EEDs were associated with food reinforcement.

### 2.5. Data Analysis

Exploratory data analysis was conducted to investigate the overall distribution of the data, and to describe them with percentages, averages, standard deviations, medians and interquartile ranges. To perform statistical analysis, we calculated the number observations of each behavior in 1-minute intervals during 30-min experimental and baseline sessions as data unity [26]. Generalized linear mixed models (GLMMs) were performed to check for significant effects of environmental enrichment in reducing stereotyped behaviors, reducing looking at people/care staff area, interaction with the enrichment device, as well as species and individual preferences. A GLMM, with a Poisson family with the sum of observations during experimental sessions, was employed to evaluate `Interacting with enrichment device (IE)’ as a response variable, species and EEDs as predictors; the individual and the experimental sessions were included in the model as random effects. The effects of age in interacting with EEDs was explored with a separate GLMM with the Poisson family, as only CSLs had different age classes in the surveyed individuals. The total number of `Interacting with enrichment device (IE)` per session was used as a response variable, and age as a predictor and experimental session/individual were used as random effects.

To evaluate the odds of presenting abnormal behaviors, a GLMM was also employed to verify the reduction in stereotypical behaviors during the session. The sum of stereotypical behavior performed during each session was used as a response variable; the EED was used as the predictor, and the individual/experimental sessions were included in the model as random effects. Similarly, a GLMM was employed to check the effects of EEDs in reducing the behavior “Looking at people/care staff area”, with the occurrences of “looking” as a response variable and the environmental enrichment as a predictor; the individual/experimental sessions were considered as random factors. Both tests employed a Poisson regression model. A total of 11 observers performed in-person/live observations to record behaviors. All of the observers were trained and passed in the interobserver agreement test (Cohen’s K = 1). Statistical analyses were performed in the R language and environment for statistical computing [27]. GLMMs were conducted using “lme4” [28] and evaluated by using “performance” [29]. Figures were constructed using ggplot2 [30] and data manipulation was performed using tidyverse [31].

## 3. Results

This study included seven California sea lions and eight northern elephant seals. Four of the CSLs were young pups at time of stranding (approximately 4 to 7 months), and were estimated to be 7–12 months at the time of the study. Three of the CSLs were yearlings (1 to 1.5 years old) at the time of stranding and older than 1.5 years at the time of the study. All of the NESs were weanlings (1.5–3 months old) at the time of stranding, and 3–6 months old at the time of study. Prior to offering the EEDs, all patients were at a point in their rehabilitation where they had access to pools, were competitively feeding for whole fish, in the weight gain period of their rehabilitation process, and at an appropriate health status to be offered and potentially benefit from EEDs. The CSLs were hospitalized for an average of 161 ± 49.5 days (min = 88, max =213). The NESs were hospitalized for an average of 93 ± 6.3 days (min = 82, max =101).

### 3.1. Baseline Behavior and Behavior in the Presence of EEDs

**Figure 2:**
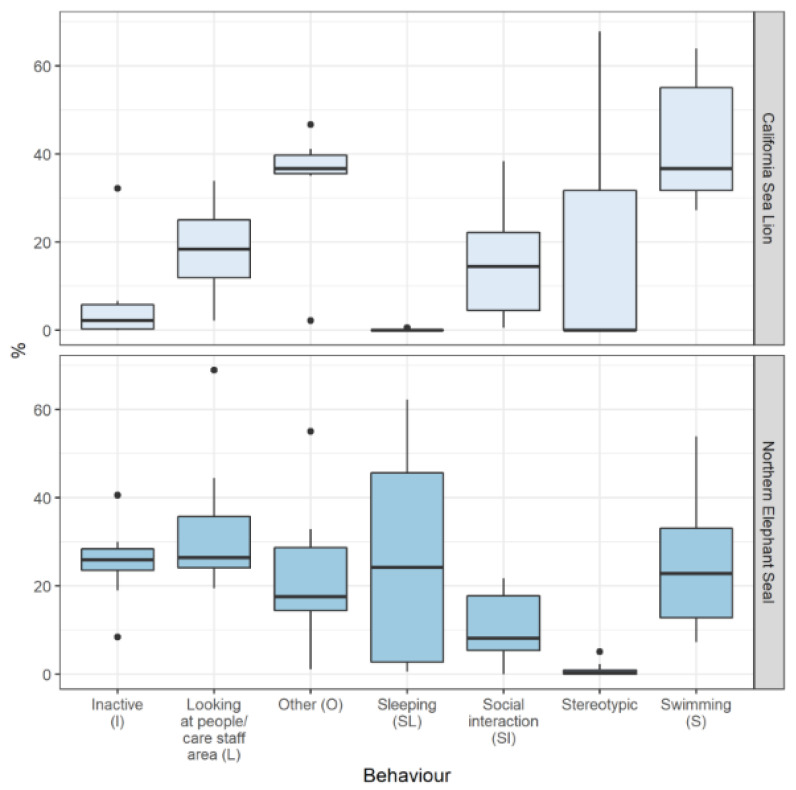
Boxplots describing medians, first quartile, third quartile (bars) and maxima and minima (whiskers) of observed percentages of exhibited behaviors by California sea lions and northern elephant seals during baseline observations. Black dots highlight outliers.

The most frequent baseline behavior exhibited by the CSLs without enrichment present was swimming, on average 31% (sd = ±7.86). The CSLs were inactive (not moving or resting on land) for 4% (sd = ±7.24). Stereotypic behavior (inappropriate suckling on self or others, or pacing on land, or pattern swimming) accounted for 17% (sd = ±24.62), and 13% (sd = ±7.01) for looking at people or staff areas. The NESs spent 18% (sd = ±12.09) swimming and 20% (sd = ±7.88) inactive when no enrichment was offered. Stereotypical behavior accounted for 0.62% (sd = ±1.11), and looking at people/staff area accounted for 24% (sd = ±9.03) (Figure 2).

Interactions with the EED were observed in most sessions. However, the CSLs Pigeon and Amber did not interact with the Wubba KONG™, and the NES Shipwreck did not interact with the Artificial Kelp. The number of interactions with the EED recorded ranged from 1 interaction for the NES Bubbles, up to 62 interactions by the CSL Skipperdee, both with Artificial Kelp (see Table 4). Overall, the number of EED interactions was 13.55 (sd ± 13.43) per session. The NESs interacted on average more with the EEDs (15.87 ± 12.32) than the CSLs (10.90 ± 14.43).

### 3.2. Species and Individual Preferences of Interacting with the EEDs

**Figure 3:**
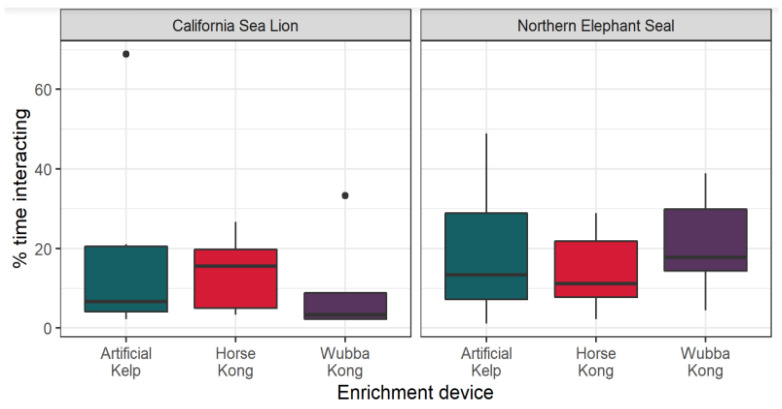
Proportional numbers of interactions (% time interacting) with EEDs per species during a 30-minute presentation session, recorded by in-person focal instantaneous sampling. Bars describe medians, first quartile, third quartile (bars) and maxima and minima (whiskers). Black dots highlight outliers.

We found significant effects of species (CSL × NES) regarding interactions with the EEDs. The NESs interacted significantly more with the EEDs than the CSLs (estimate = −1.2728 SE = 0.2778; Z = 6.; *p* < 0.0011) (Figure 3). However, within a species, we did not find significant differences among the use of EEDs (Artificial Kelp, Horse Kong and Wubba Kong). Regarding individual preferences, the NESs Barracuda (estimate = −1.96869; SE = 0.90344; Z = −2.179; *p* = 0.02), Bubbles (estimate = −2.7517; SE = 1.0468; Z = −2.629; *p* = 0.008), Flower (estimate = −1.8234; SE = 0.8869; Z = −2.056; *p* = 0.039) and Shipwreck (estimate = −2.7534; SE = 1.0471; Z = −2.630; *p* = 0.008) demonstrated less interest for Artificial Kelp; Bubbles also had fewer interactions with the Horse Kong (estimate = −3.4184; SE = 1.2579; Z = −2.718; *p* = 0.006) and Wubba Kong (estimate = −2.6771; SE = 1.0341; Z = −2.589; *p* = 0.009); Flower had significantly fewer interactions with the Wubba Kong (estimate = −1.8234; SE = 0.8869; Z = −2.056; *p* = 0.039).

**Figure 4:**
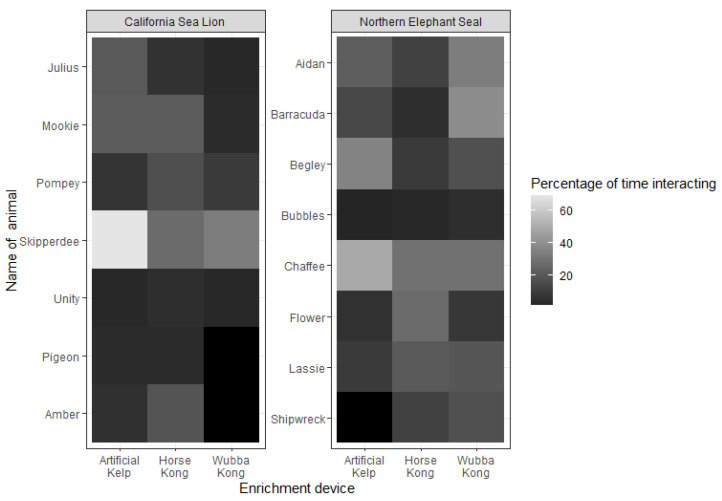
Heatmap of the percentages of individual use of environmental enrichment devices per species during presentation sessions. Black = no interaction.

Overall, the CSLs preferred the Horse KONG^™^ and the NESs preferred the Wubba KONG^™^. More detailed metrics of the proportions of use per enrichment type, per species, including preferences per individual, are captured in the heat map (Figure 4).

### 3.3. Effects of Age on Use of EEDs

In this study, all of the CSLs were pups or yearlings. A GLMM was performed for the interaction with EEDs, considering the age class for CSLs. This group was composed of three individuals categorized as yearlings (Amber, Pigeon and Unity). The remaining four individuals were categorized as pups. We observed that age class had significant effects on the amount of use of the EEDs. The CSL yearlings interacted significantly less compared to the pups, without distinction of the EED used (intercept = 1.1105; estimate = −1.9182; SE = 0.4741; *p* < 0.0001). All of the elephant seals were weanlings/pups.

### 3.4. Effects of EEDs on Stereotypical Behavior

None of the NESs displayed stereotypical behavior during any of the sessions. Three of the seven CSL exhibited stereotypical behaviors as defined earlier in this study (Amber, Julius and Pigeon), including suckling, pacing and pattern swimming.

**Figure 5:**
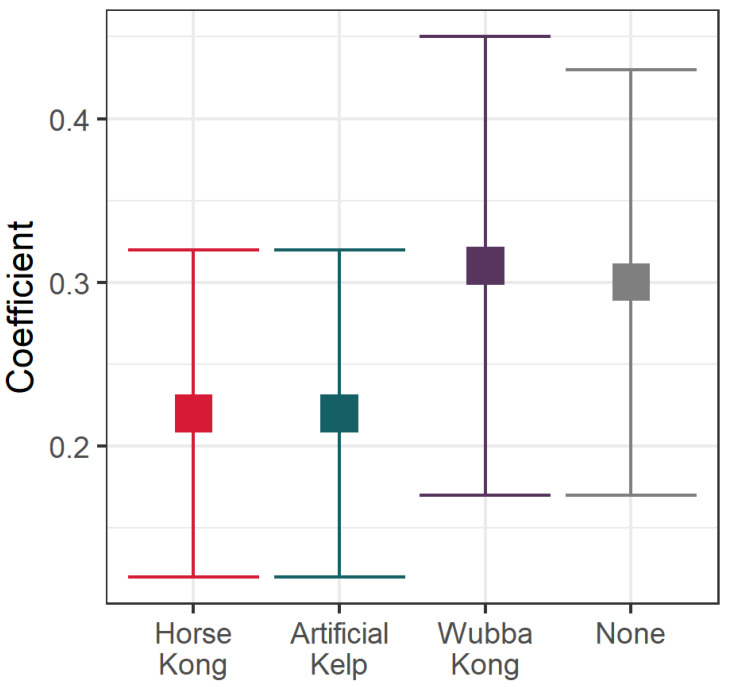
Coefficients from a Poisson GLMM of California sea lions (CSL) displaying stereotypic behaviors in relation to the environmental enrichment device (EED) presented.

A GLMM was used on data from these three patients to evaluate the reduction in stereotypic behavior in a binomial model. A significant reduction in stereotypical behavior was observed during experimental sessions compared to no enrichment treatments. The EEDs with a significant effect in reducing abnormal behaviors in CSLs were the Horse KONG^™^ (estimate = −0.2964; SE = 0.1425; Z = −2.080; *p* < 0.03749) and Artificial Kelp (estimate = −0.3218; SE = 0.1555; Z = −2.; *p* = 0.03857). Although the CSLs showed interest in and had interactions with the Wubba KONG^™^, the Wubba KONG^™^ did not have a significant impact on the prevalence of stereotypical behavior (estimate = 0.0294; SE = 0.1257; Z = 0.234; *p* = 0.8151). Our results show that the Horse KONG^™^ and Artificial Kelp were the most effective EEDs for reducing the probability of displaying stereotypical behaviors in CSLs (Figure 5).

### 3.5. Effects of EEDs on Looking at People/Care Staff Areas

**Figure 6:**
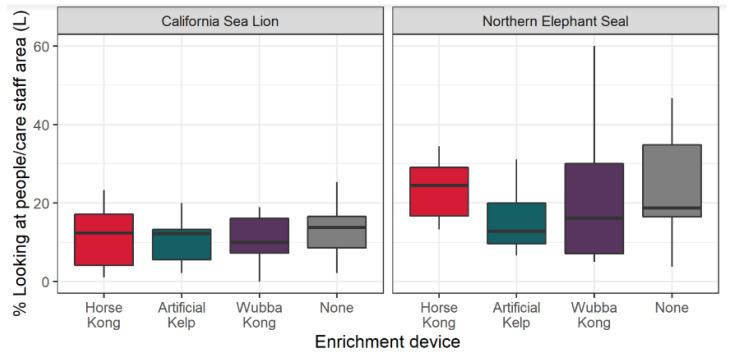
Boxplot describing medians, first quartile, third quartile (bars) and maxima and minima (whiskers) of percentages of time spent looking at people/care staff area during presentation sessions of environmental enrichment devices (Artificial Kelp, Horse Kong^™^ and Wubba Kong^™^), and with no EEDs present (“none”).

A GLMM was employed to evaluate the effects of EEDs on animals gazing towards people and care staff areas. We found a difference between CSLs and NESs when EEDs were used, where NESs gazed significantly more when compared to CSLs under effects of EEDs (estimate = 0.6607; SE = 0.2283; Z = 7.286; *p* = 0.003). The use of the Artificial Kelp had significant effects in reducing the behavior “looking at people/care staff area” (estimate = −0.3774; SE = 0.0868; Z = −4.346; *p* < 0.0001), as did the Wubba Kong™ (estimate = −0.2289; SE = 0.07897; Z = −2.899; *p* = 0.003). Although the Horse Kong reduced the behavior “looking at people/care staff area”, no significant effect was found (estimate = −0.04737; SE = 0.0742; Z = −0.637; *p* = 0.5238). The behavior recorded under no influence of EED (i.e., none) was employed as an intercept (estimate = −1.25161; SE = 0.1717; Z = 7.286; *p* < 0.0001). Therefore, the use of the Wubba KONG™ as with the Artificial Kelp tested in this study reduced the likelihood of individuals expressing undesired looking behavior (see Figure 6).

## 4. Discussion

Offering EEDs to CSL pups/yearlings and NES weanlings, while undergoing rehabilitation, was an effective way to decrease stereotypical behaviors in CSLs, and helped minimize the risk of habituation by decreasing the time these wild CSLs and NESs looked in the direction of human caregivers and staff areas. Marine mammal rehabilitation centers are inherently less stimulating than the animals’ natural marine environment, and risk animals becoming habituated to people due to the increased exposure and close contact to humans compared to a wild setting. Unwanted behaviors included staring at people, which promotes habituation to humans. Daily care activities, e.g., cleaning and feeding, and the association of human caretakers with food results in animals paying attention to care staff and staff-related areas, and engaging in staring and anticipatory behaviors [32,33,34,35]. This can not only result in an increased risk of habituation [36], but also cause unintentional stress, encourage the development of stereotypical behaviors, and may negatively impact their post-release success [37,38,39]. This study shows that offering EEDs to young CSLs and NESs during rehabilitation has the potential to redirect and reduce these behaviors, which may improve the welfare of pinnipeds during rehabilitation, as well as contribute to a more successful outcome following their release back to the wild. The Horse Kong^™^ and Artificial Kelp were particularly successful in reducing stereotypic behaviors. We speculate that it was possibly due to the affordances they have, both partially or fully submerged underwater; the bite ring on the Kong allowed the object to be dragged under the water, and in the case of the Artificial Kelp, it was large enough for social play and interaction. Despite possessing elements of both other types of enrichment, the Wubba Kong was less effective at reducing the prevalence of stereotypic behaviors; this may have been due to the size of the device not being large enough for social play, or the fabric may have not been preferable for the animals in encouraging functional behaviors. Both the Wubba Kong^™^ and Artificial Kelp did reduce the likelihood of individuals expressing undesired looking behavior towards the staff areas; this is perhaps because of the similarities of long strips for interactive and social behavior, as well as the Artificial Kelp being larger and underwater, and the Wubba Kong^™^ being smaller and more agile on the surface; all of these factors contributed to the reduction in this undesired behavior.

Capturing the nature of these differences in an objective manner was beyond the scope of this study, and presents an opportunity for future research into the EED preferences of pinnipeds and the functional value of EEDs offered.

All three EEDs assessed in this study (Artificial Kelp, Horse KONG^™^ and Wubba Kong^™^) were deemed safe for use. Both species interacted with all three EEDs. Between the two species, there were no specific preferences amongst the three EEDs; however, there were strong individual preferences within a species. This is consistent with findings in other species such as dolphins [40] and other taxa [41,42]. These findings suggest that to maximize EED engagement, enrichment programs should aim to tailor to each animal’s preferences whenever possible, while continuing to offer a variety of enrichment to gauge and maintain interest in novel items. Providing multiples of the same EED would be ideal when similar preferences are observed within a group of animals so that all individuals may interact with their preferred device. Anecdotally, some animals were slower and more hesitant to approach and interact with the EEDs, while others quickly investigated and interacted with the EEDs with little reservation. 

Apart from individual preferences, differences in EED interactions in the CSL group may have been associated with developmental stages at the time of stranding (pup vs. yearling) and/or underlying disease contributions which may have influenced their ability and interest to engage with the EEDs. It is understood that age may have an influence on preference and engagement with enrichment devices in marine mammals [40]; however, the nuances of this impact at a month-to-month developmental level for very young animals is not clearly understood. For example, we suspected that early maternal separation when a pup is still nursing may have resulted in a propensity for developing stereotypical suckling behavior compared to other pups that were slightly older at the time of stranding. Julius, a male sea lion pup estimated to be 4 months old at the time of stranding, immediately displayed habitual suckling behavior on himself and other pups; this persisted throughout his rehabilitation, with a subjectively reduced frequency in the month prior to release (at approximately 9 to 10 months old). The results of the present study showed Julius’ suckling behavior significantly decreased when EEDs were present, compared to when no EEDs were offered. Interestingly, when engaged with the EEDs, no animals were observed suckling on the EEDs, but instead engaged in play interactions with the devices. Further investigation needs to be carried out to better understand the positive impact EEDs can provide younger pinniped patients, and when/how EEDs should be introduced into the rehabilitation setting. Habitual suckling can result in aerophagia (swallowing of air), which can cause abdominal discomfort and ileus (a disruption of the normal propulsive ability of the intestine) in these malnourished pups with significantly compromised gastrointestinal tracts. In addition, habitual sucklers can latch onto ears, wounds, pox lesions and prepuces, resulting in tissue damage, transmission of bacterial and viral infections, and the potential for ascending urinary tract infections. Thus, we suspect that incorporating EEDs in young pup rehabilitation may significantly decrease the risk of secondary morbidities and decrease rehabilitation time.

Amber was a male yearling CSL who presented with a chronic left eye injury (*phthisis bulbus*), resulting in the animal being non-visual in his left eye. Amber displayed stereotypical pattern swimming and pacing behavior during his time in rehabilitation. The unilateral blindness resulted in a decreased field of view, which was evident in the way he swam and angled his head with his visual eye in the direction of interest when trying to investigate the EEDs, and during feedings and interactions with the environment. This may have contributed to Amber being slightly more hesitant to interact with the EEDs, and being the least interested in the Artificial Kelp. Findings from other species indicate that various disabilities may impact an animal’s ability to engage with enrichment; for example, blind black bears in one study tended to interact with foraging enrichment less than sighted bears [43]; Amber’s case study presents evidence that the same may be true for pinnipeds, which have sophisticated vision systems and may struggle to find objects when eyesight is impaired [44]. In this case, we suggest that our results demonstrate that there is a potential unique value of utilizing EEDs in the rehabilitation process for patients that are learning to adapt to permanent injuries, particularly for eye injuries which can be debilitating [44,45]. More research should be conducted to better understand the methods in which EEDs can be specifically tailored for individual wild animals that must learn to adapt to new limitations, which may improve their outcomes following release.

Pigeon was a yearling female CSL with severe muscle wastage on presentation due to a protozoal Sarcocystis infection. She was paraparetic in all limbs, leading to scooting on her chest when attempting to ambulate, which improved with treatment. She was ambulating and swimming normally at time of the EED study. Of all the CSLs, Pigeon was the one that interacted least with the EEDs offered. This may have been the result of muscle fatigue associated with recovery from her myopathy; lower energy levels, in general, may have led to a lack of engagement with the EEDs [43]. Alternatively, her age class may have influenced her engagement with the EED devices, as two yearling CSLs, Unity and Amber, also interacted less with the EEDs compared to the younger sea lion pups; this would be consistent with age differences in enrichment engagement seen in dolphins [40]. While initially avoiding EEDs when pattern swimming, she became interactive with the devices to a level of eliminating the pattern swimming all together as the study progressed. A reduction in pattern swimming under EED conditions is consistent with findings from other pinnipeds [6,38,46] and in other marine mammals, including dolphins [47]. In Pigeon’s case, offering the EEDs may have encouraged her to swim and play more while interacting with them, which likely provided helpful physical therapy she may not have otherwise engaged in during her rehabilitation process if the EEDs were not offered. Again, this shows the value of offering tailored EEDs to pinnipeds in rehabilitation, and how they may improve recovery and long-term outcomes of patients following release.

The most reported behavioral concern in hospitalized marine mammals is fixed pattern swimming [6,36] and suckling on self or cohorts, as observed with pups. Self-directed and injurious behaviors such as flipper chewing, excessive self-grooming and body-scratching against hard surfaces have also been reported in pinnipeds [38,39]. These behaviors are widely accepted as signs of stress [48]. These behaviors may increase and become associated with animal caregivers, as they are feeding the patients several times a day, which in turn may increase looking at the staff or staff areas. While visual barriers can be of assistance to reduce looking behaviors, the ability to have a view must be considered and the behavior closely monitored. Blocking a view sometimes increases an animal’s drive to see what is going on in their environment, and hearing activities or for other animals without the ability to see what is goin on may cause stress (personal observation Brando). It is also important to provide opportunities to engage in natural behaviors such as play and explore promoting species-specific and social behaviors [49]. 

This study demonstrates that EEDs can encourage behaviors such as exploration and play in a rehabilitation setting. Interactions with EEDs were characterized by explorative behaviors, such as manipulating and investigating the devices; interactions with enrichment devices are generally seen and experienced as play, and can assist in learning during this important developmental life stage. EEDs can also be important for learning other skills, including socially acquired ones; most young marine mammals acquire appropriate behaviors from age-matched cohorts and/or mothers [50], making them the ones that benefit more from access to EEDs. Studies also showed that immature animals are more motivated to try novel tasks and challenges, which make them good candidates for using enrichment devices in rehabilitation and reintroduction [51]. Further study on the specific behavioral interactions with different enrichment types and the intricacies of how different objects are used and played with will be beneficial to understanding the specific behavioral lessons learned from EEDs.

When the elephant seals were inactive (resting or not moving) or visibly sleeping, they had no interest in interacting with any of the enrichment devices. It has long been known that sleep and rest are important for healing [52], restoration, and overall wellbeing in humans and other animals, and that differences in sleep and rest behaviors exist between species. Therefore, we chose not to disturb or expect these convalescing animals to interact with the EEDs. Inactive and sleeping behavior was not significantly impacted by the presence of EEDs, which is consistent with findings in harbor seals and gray seals (6). Enriching animals is important, but considering their sleep and rest, and the impact of other activities throughout their day is important as well. While the NESs did not show interest in interacting with the EEDs while sleeping and inactive on land, the NESs were more interested in interacting with the EEDs than the CSLs when not resting. To the authors, this was one of the more surprising findings of this study, as NESs are generally perceived as less active, and spending much of their time being inactive and sleeping (personal observation, Donald, Walters).

Most EEDs used with marine mammals are floating objects [53], rather than being neutrally buoyant in the water column, or negatively buoyant in sinking to the bottom of a pool. In the context of rehabilitation, the challenge EEDs provide is ideally relevant for post-release success, intrinsically motivating, and possible to master, e.g., such as diving, breath holding and apprehending objects with animals’ mouths. Floating toys catch an animal’s interest immediately, but subjects may quickly become less interested in them [54]. Thus, by providing EEDs which sink to the bottom, such as the Horse KONG^™^, and including a combination of parts that float at the surface while also hanging in the water column such as the Artificial Kelp and the Wubba KONG^™^, we were able to provide different opportunities from the surface to the bottom of pools.

While the sample size was small, the findings of this study offer simple and practical options for marine mammal rehabilitation centers to reduce the prevalence of stereotypic behaviors and habituation to humans in patients, while encouraging cognitive and physiological development, expediting recovery times, and potentially improving success rates in survival following release of patients back into their natural habitat [16,18]. Future studies would benefit from focusing on specific outcomes of EEDs in rehabilitation programs, such as early intervention with enrichment to reduce suckling, anxiety and habituation in CSL pups, as well as looking at whether providing enrichment encourages natural behaviors relevant to survival, such as foraging and navigating social dominance, during critical developmental stages [55]. Research in rehabilitation centers aligns with the objectives of stranding networks: “At its best, stranding response combines welfare, science, and conservation priorities resulting in humane response to live stranded marine mammals and quality data collection and analyses that inform conservation and management strategies” [56].

## 5. Conclusions

Determining how EEDs influence animal cognition can help better elucidate the benefits of EEDs in wildlife rehabilitation settings, as well as make a difference in a patient’s success following reintroduction to the wild [51]. Wildlife rehabilitation hospitals are faced with the need to balance the benefits of time needed for treatment with the potential impact that prolonged human care can exert in terms of altering natural/wild behaviors necessary to survive independently in the wild [55]. In addition, there may be risk of delayed or inappropriate behavioral development in pups undergoing rehabilitation due to not being exposed to the natural stimuli found around the rookeries. We propose that even if animals have different medical challenges when arriving in a rehabilitation center, as soon as they are healthy enough and it is safe, a variety of enrichment or more dynamic habitats should be available to provide opportunities to encourage and develop natural behaviors such as exploration, diving and swimming.

While some individual differences were found regarding the interest in specific EEDs, the results generally show that environmental enrichment was suitable and beneficial for all CSLs and NESs involved in this study. All EEDs offered during the study at the PMMC reduced stereotypic behavior in CSLs and reduced leading indicators of habituation in both CSLs and NESs. Different preferences, personalities and age classes influence studies in ways that are difficult or impossible to control in an experimental setting. However, the outcomes in this study highlight the fundamental and beneficial aspect of EEDs for young pinnipeds in rehabilitation centers as a method of improving their quality of life. The addition of environmental enrichment to increase positive animal welfare is one of the steps that can inspire improved animal welfare standards in rehabilitation centers globally.

## Figures and Tables

**Table 1:** Name, sex, age and species of animals in the present study.

Species	Name	Sex	Estimated Age and at Study	Notes
California Sea Lion	Amber	Male	Yearling: approx. 1–1.5 years and older than 1.5 years	Unilateral blindness Performing stereotypic behaviors
	Julius	Male	Pup: approx. 4 to 7 months and 7–12 months	Performing stereotypic behaviors
	Mookie	Female	Pup: approx. 4 to 7 months and 7–12 months	
	Pigeon	Female	Yearling: approx. 1–1.5 years and older than 1.5 years	Performing stereotypic behaviors
	Pompey	Female	Pup: approx. 4 to 7 months and 7–12 months	
	Skipperdee	Female	Pup: approx. 4 to 7 months and 7–12 months	
	Unity	Male	Yearling: approx. 1–1.5 years and older than 1.5 years	
Northern Elephant Seal	Aidan	Female	Weaners: 1.5–3 months and 3–6 months	
	Barracuda	Female		
	Begley	Female		
	Bubbles	Male		
	Chaffee	Female		
	Flower	Female		
	Lassie	Female		
	Shipwreck	Male		

**Table 2:** Behavioral definitions.

Behavior	Definition
Swimming (S)	Animal is moving in the water, not interacting with anything or any other animal.
Interacting with Enrichment Device (IE)	Animal is interacting, exploring or playing with an enrichment device in some way (e.g., touching, biting, smelling), whether in the pool or on land.
Social Interaction (SI)	Animal is interacting in some way with another animal in the pool or on land.
Stereotypic Behavior (ST)	Animal is repeating a behavior with no apparent function (e.g., pattern swimming, suckling on self, sliding in and out of the water)
Sleeping (SL)	Animal has eyes closed, breathing slowed and is inactive.
Inactive (I)	Animal is not moving, but not sleeping; can see that the animal is awake, just not active.
Looking at People/Care Staff Area (L)	Animal is angling head and gaze towards nearby humans or towards care staff areas.
Other (O)	Behavior not listed on the ethogram.

**Table 3:** Number of sessions per patient in the study and enrichment device.

	California Sea Lions (Number of sessions)								
Enrichment	Amber	Julius	Mookie	Pigeon	Pompey	Skipperdee	Unity	Total number of sessions	
Artificial Kelp	2	2	3	2	2	3	3	17	
Horse KONG^™^	2	3	3	3	3	3	3	20	
Wubba KONG^™^	2	3	3	3	3	3	3	20	
None	6	8	9	8	8	9	9	57	
Enrichment	Northern Elephant Seals (Number of sessions)								
	Aidan	Barracuda	Begley	Bubbles	Chaffee	Flower	Lassie	Shipwreck	Total number of sessions
Artificial Kelp	3	3	3	3	3	3	3	3	24
Horse KONG^™^	3	3	3	3	3	3	3	3	24
Wubba KONG^™^	3	3	3	3	3	3	3	3	24
None	9	9	9	9	9	9	9	9	72

**Table 4:** Percentages of behaviors expressed by California sea lions and northern elephant seals during the presentation of environmental enrichment devices (Wubba Kong ^™^, Horse Kong ^™^ and Artificial Kelp).

Behaviors	California Sea Lions			Northern Elephant Seals		
	Artificial Kelp	Horse KONG^™^	Wubba KONG^™^	Artificial Kelp	Horse KONG^™^	Wubba KONG^™^
Swimming (S)	27.65	31.72	33.17	11.25	17.20	9.10
Interacting with enrichment device (IE)	20.39	13.36	7.50	16.94	13.73	21.74
Social interaction (SI)	4.71	9.18	11.17	2.08	5.27	3.13
Stereotypic	9.80	10.35	14.50	0.00	0.83	1.49
Sleeping (SL)	0.00	0.00	0.00	20.28	11.37	16.58
Looking at people/care staff area (L)	9.61	1185	11.33	15.56	23.44	18.89
Inactive (I)	6.86	6.18	0.83	19.72	11.93	14.67
Other (O)	20.98	17.36	21.50	14.17	16.23	14.40

## Data Availability

Not applicable.
